# Complete Plastid and Mitochondrial Genomes of *Aeginetia indica* Reveal Intracellular Gene Transfer (IGT), Horizontal Gene Transfer (HGT), and Cytoplasmic Male Sterility (CMS)

**DOI:** 10.3390/ijms22116143

**Published:** 2021-06-07

**Authors:** Kyoung-Su Choi, Seonjoo Park

**Affiliations:** 1 Institute of Natural Science, Yeungnam Univiersity, Gyeongsan-si 38541, Gyeongbuk-do, Korea; choiks010@gmail.com; 2 Department of Life Sciences, Yeungnam University, Gyeongsan-si 38541, Gyeongbuk-do, Korea

**Keywords:** *Aeginetia indica*, Orobanchaceae, plastid, mitogenome, intracellular gene transfer (IGT), horizontal gene transfer (HGT), cytoplasmic male sterility (CMS)

## Abstract

Orobanchaceae have become a model group for studies on the evolution of parasitic flowering plants, and *Aeginetia indica*, a holoparasitic plant, is a member of this family. In this study, we assembled the complete chloroplast and mitochondrial genomes of *A. indica*. The chloroplast and mitochondrial genomes were 56,381 bp and 401,628 bp long, respectively. The chloroplast genome of *A. indica* shows massive plastid genes and the loss of one IR (inverted repeat). A comparison of the *A. indica* chloroplast genome sequence with that of a previous study demonstrated that the two chloroplast genomes encode a similar number of proteins (except *atpH*) but differ greatly in length. The *A. indica* mitochondrial genome has 53 genes, including 35 protein-coding genes (34 native mitochondrial genes and one chloroplast gene), 15 tRNA (11 native mitochondrial genes and four chloroplast genes) genes, and three rRNA genes. Evidence for intracellular gene transfer (IGT) and horizontal gene transfer (HGT) was obtained for plastid and mitochondrial genomes. ψ*ndhB* and ψ*cemA* in the *A. indica* mitogenome were transferred from the plastid genome of *A. indica*. The *atpH* gene in the plastid of *A. indica* was transferred from another plastid angiosperm plastid and the *atpI* gene in mitogenome *A. indica* was transferred from a host plant like *Miscanthus siensis*. *Cox2* (*orf43*) encodes proteins containing a membrane domain, making ORF (Open Reading Frame) the most likely candidate gene for CMS development in *A. indica*.

## 1. Introduction

The structure and gene contents of plastid genomes are highly conserved in most flowering plants and range from 110 to 160 kb in length and contain 110 genes (~79 protein coding, 29 tRNA, and 4 rRNA genes) [1]. In contrast, angiosperm mitogenomes are remarkably divergent in size, structure, and mutation rate. Most angiosperm mt genomes contain 24 to 41 protein coding genes, three rRNA genes, and two or three rRNA genes [2,3,4,5,6,7]. Recent mitochondrial genome studies have demonstrated horizontal gene transfer (HGT) and intracellular gene transfer (IGT) [5,8,9,10,11]. Additionally, plant mitochondria contain mitochondrial-encoded cytoplasmic male sterility (CMS) genes, which are related to the production of functional pollen or functional male reproductive organs [12]. Also, CMS genes affect the evolution of the mitochondrial genome by influencing mitochondrial recombination and rearrangement [13,14,15,16].

Orobanchaceae is a family of mostly parasitic plants of the order Lamiales and contains about more than 2000 species in 90–115 genera. Members of this family include all types of parasitic plants such as hemiparasites and holoparasites [17,18,19]. Holoparasites (obligate parasites) cannot live without a host, whereas hemiparasites (facultative parasites) can. The plastomes of Orobanchaceae (parasite species) are remarkably variable with respect to genome size, genome structure, and gene contents. The majority of photosynthesis-related and plastid-encoded NAD(P)H-dehydrogenase (NDH) complex genes in the Orobanchaceae plastome have been lost or pseudogenized [20,21], and in several species, one IR (inverted repeat) copy has been completely lost, and as a result, Orobanchaceae plastomes range from 45 kb (*Conopholis americana*) to 160 kb (*Schwalbea americana*) in length [22]. The complete mitochondrial genome of Orobanchaceae, *Castilleja paramensis* has been reported [23], and Zavas et al. [24] reported the mitochondrial genes of two *Lathraea* species. Genes of the Orobanchaceae plastome and mitogenome, such as *atp1* [25], *rpoC2* [26], *atp6* [27], and *nad1* [28], exhibit several transfers between Orobanchaceae and angiosperms.

*Aeginetia indica* is a holoparasitic plant of the Orobanchaceae family and is parasitic on the roots of monocots like *Miscanthus* [18]. Previous phylogenetic studies have shown that *A. indica* is united with *Stiga*, *Buchera*, *Radmaea,* and *Harveya* [29,30]. The plastome of *A. indica* has been reported to be 86,212 bp in size and to have lost almost all photosynthesis-related genes [31].

The present study was undertaken to determine the plastome and mitogenome of *A. indica* and to compare these with previously reported results [31], especially with respect to mitogenome size, gene, and intron contents and repeats, and to analyze the HGT, IGT, and CMS genes in the *A. indica* mitogenome.

## 2. Results and Discussion

### 2.1. Characteristics of the A. Indica Plastid Genome

**Figure 1:**
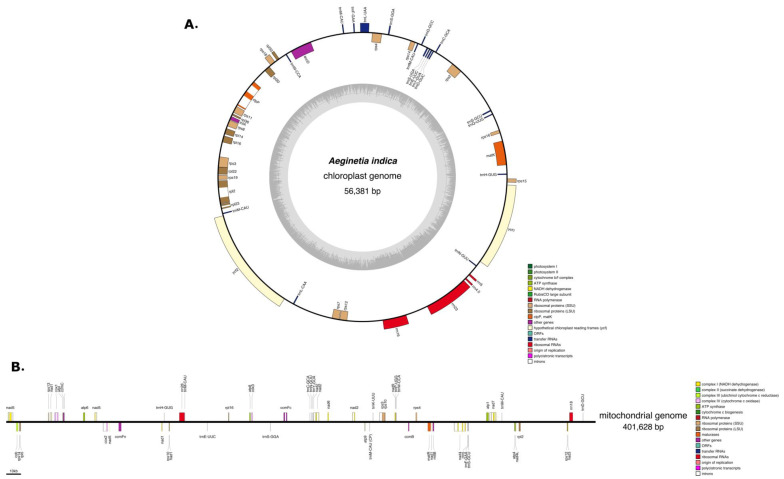
(**A**) Chloroplast genome of *Aeginetia indica*. (**B**) Mitochondrial genome of *A. indica*. Top and bottom genes are transcribed in forward and reverse directions, respectively.

A previous study [31] showed that the plastid genome of *A. indica* is 86,212 bp in length with an LSC (Large single Copy), SSC (Small Single Copy), and two IRs. However, we found the complete plastid genome of *A. indica* (GenBank accession number: MW851293) is 56,381 bp in length and contains an LSC, SSC, and only one IR (Figure 1) together with 26 protein coding genes.

The coverage of *A. indica* in the present study was 6089X (Figure S1). In contrast, the coverage of the plastid in the previous study of *A. indica* had gaps and low coverage values (Figures S1 and S2). Furthermore, the GC content of the plastid genome in this study (32.9%) was higher than in the previous study (34.4%), and 18 tRNAs was smaller in the present study. Protein coding gene contents in the plastid genome of *A. indica* showed all *atp, ndh, psa, psb, pet,* and *rpo* gene groups have been lost together with *cemA, ccsA, rbcL, ycf3,* and *ycf4* genes. However, the *ndhB* gene was pseudogene and the *atpH* gene remained intact (Table 1).

The *A. indica* plastid genome is the second smallest among the Orobanchaceae, in which previously sequenced genomes ranged in size from 45,673 bp in *Conopholis americana* (NC_023131) to 160,910 bp in *Schwalbea americana* [22]. Wicke et al. [22] showed that 16 protein genes (*matK, rpl2, rpl16, rpl20, rpl33, rpl36, rps11, rps2, rps4, rps7, rps12, rps14, rps18, rps8, ycf1,* and *ycf2*), 15 tRNAs (*trnD-GUC, trnE-UUC, trnfM-CAU, trnH-GUG, trnI-CAU, trnL-UAG, trnM-CAU, trnN-GUU, trnP-UGG, trnQ-UUG, trnS-GCU, trnS-UGA, trnW-CCA*, and *trnY-GUA*), and four rRNAs (rrn16, rrn23, rrn4.5 and rrn5) are present in the chloroplast genomes of Orobanchaceae and that *A. indica* also contains these genes. Most of the chloroplast genome lengths of hemiparasites in Orobanchaceae are longer than holoparasites in Orobanchaceae (Figure S3). Hemiparasitic species contain pseudogenes of photosynthesis-related genes and NADH dehydrogenase complex (*ndh* genes) and only a few genes have been lost. However, many genes in holoparasitic species have been completely lost. Especially in *A. indica*, most of photosynthesis-related genes and *ndh* genes have been completely lost and have one IR region.

We conducted phylogenetic analysis using a gene data matrix based on 14 protein coding genes from 34 species (Table S1) with 13,499 bp aligned nucleotides. Orobanchaceae species formed a monophyletic group with high bootstrap values, except for *P. cheilanthifolia*. Two *A. indica* formed a highly supported clade (Figure S3). It is possible that the previous study [31] and the present study were performed on different species *A. indica* and that reclassification of the genus *Aeginetia* is required.

### 2.2. Characteristics of the A. Indica Mitogenome

**Figure 2:**
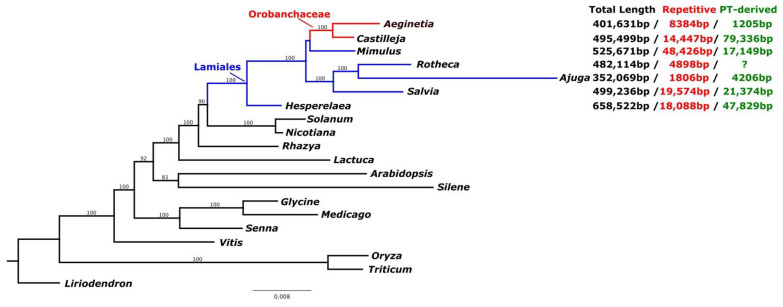
Cladogram of species included in the study. Topology was based on the ML tree generated concatenated MT genes listed in Table S6. Genome size, amount of plastid-like and repetitive DNA in seven Limiales species. Blue branches and red branches indicate Lamiales and Orobanchaceae species, respectively.

The assembled *A. indica* mitogenome was 491,631 bp long (GenBank accession number: MW851294) with a GC content of 43.5% (Figure 1). The average coverage of the *A. indica* mitogenome was 1379.9X (Figure S4). We could not assemble a circular mitochondrial genome for *A. indica* and considered that the genome might be linear or a collection of sub-genomic molecules that arise via recombination of repeat regions [32]. Tandem repeats ranged in length from 37 to 419 bp with a total length of 8384 bp. We identified 12 chloroplast genome fragments in the mitochondrial genome that included genes and intergenic regions (Figure 1 and Figure 2, Table S2). The fragments ranged from 57 to 154 bp. The mitochondrial genome contained a pseudogene of *ndhB*, a partial *rps4* gene, six tRNAs, and two IGS (Intergenic spacer) regions. The *A. indica* mitochondrial genome is common in terms of genome and repetitive sequence sizes. However, the *A. indica* mitochondrial genome had a smaller plastid-derived sequence size than other Lamiales (Figure 2). A total of 34 complete native mitochondria protein coding genes and one complete chloroplast protein coding gene (*atpI*) were annotated in the mitogenome with 15 tRNAs (11 native mitochondrial tRNAs and four plastid-derived tRNAs) and three rRNAs (Figure 1, Tables S3–S5). The *A. indica* mitochondrial genome did not contain ribosomal protein subunit genes (*rps1, rps2, rps7, rps11*, and *rps19),* and two respiratory genes (*shd3,* and *sdh4)*, which have been lost in angiosperms (Table S3) [14,23,33,34]. In previous studies, ribosomal protein gene (*rps10* and *rps7*) and *sdh* (*sdh3* and *shd4*) genes were functionally transferred to the nuclear genome many times [34,35]. Similarly, the ribosomal genes and *sdh* genes in *A. indcia* were also transferred to the nuclear genome.

### 2.3. IGT and HGT of A. Indica Organelle Genomes

Angiosperm genomes sometimes contain foreign genes caused by IGT and/or HGT. In plants, IGT between cp, mt, and nuclear genomes is a common and well-known evolutionary phenomenon [36,37,38,39,40]. In the Orobanchaceae species, most chloroplast genes and fragments have been transferred from the nuclear or mitochondrial genomes of chloroplasts [41].

**Figure 3:**
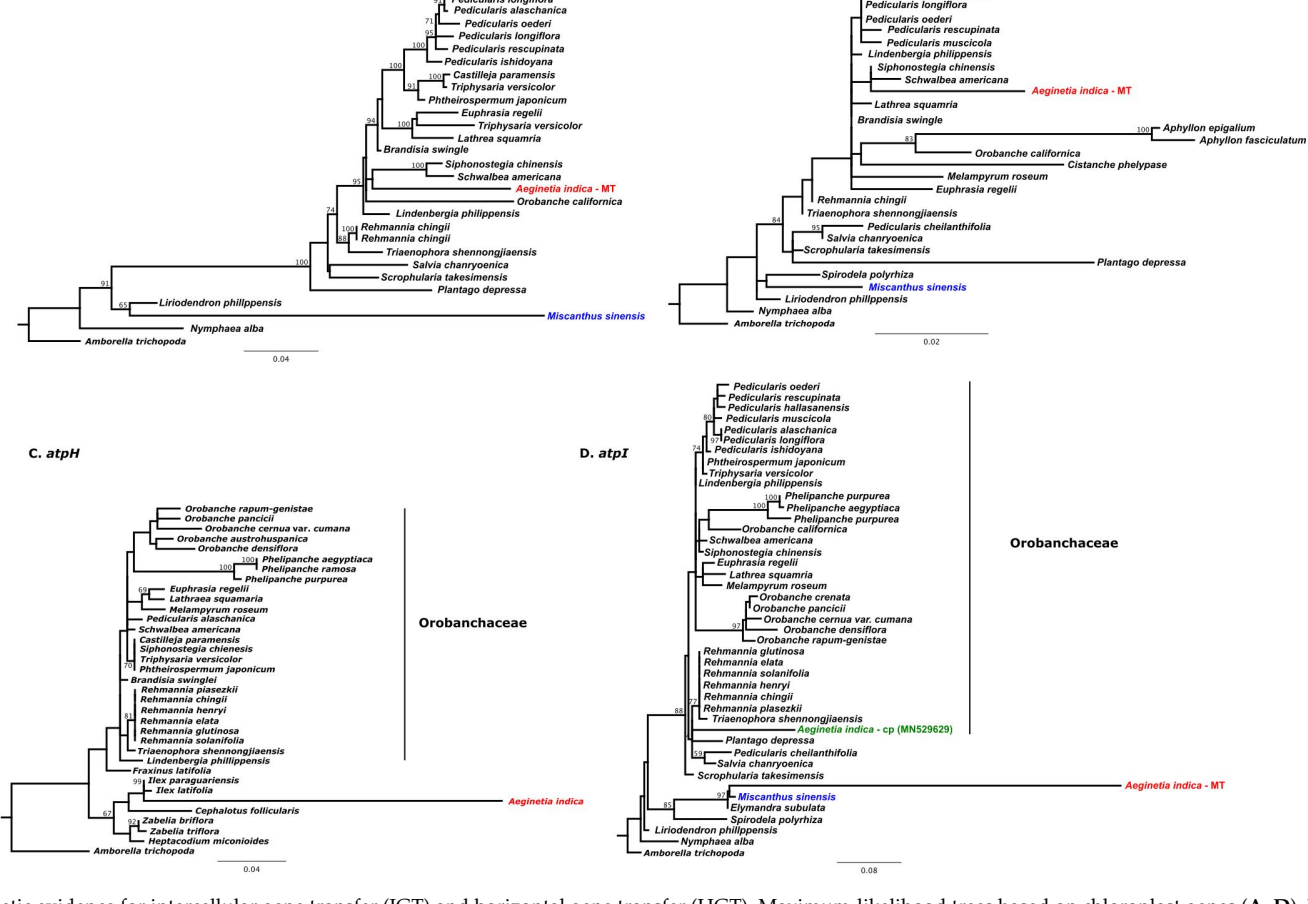
Phylogenetic evidence for intercellular gene transfer (IGT) and horizontal gene transfer (HGT). Maximum-likelihood trees based on chloroplast genes (**A**–**D**). Bootstrap values of > 60% are shown on branches. (**A**) IGT of fragment of the *cemA* gene in the *A. indica* mitogenome (**B**) IGT of fragments of the *ndhB* gene in the *A. indica* mitogenome (**C**) HGT of *atpH* in the *A. indica* plastid genome (**D**) HGT of *atpI* in the *A. indica* mitochondrial genome. Colors indicate *A. indica* (red) and *Miscanthus sinensis* (blue).

To identify the genes transferred between the chloroplast and mitochondrial genomes of *A. indica*, we used BLAST analysis to identify sequences with significant homology in the two genomes. In was reported in the previous study [31] that most chloroplast genes of *A. indica* could not be detected in its transcriptomes, which suggested that they were non-functional [31]. We detected two pseudogenes (ψ*ndhB* and ψ*cemA*) of chloroplast genes in the *A. indica* mitochondrial genome (Figure 3A,B). Phylogenetic analyses of these two pseudogenes showed that both are monophyletic groups with Orobanchaceae species (Figure 3A,B). Thus, we suggest that the ψ*ndhB* and ψ*cemA* genes in the *A. indica* mitochondrial genome were transferred from the *A. indica* chloroplast genome and are probably the result of IGT. Cusimano and Wicke [41] suggested that most of the photosynthesis-related genes lost from Orobanchaceae chloroplast genomes have been transferred to mitochondrial or nuclear genomes by IGT and subsequently fragmented.

In a previous study [31], it was reported that the *atpH* gene in the *A. indica* chloroplast genome had been lost. However, we found an intact *atpH* gene in the *A. indica* chloroplast genome that is not typically found in Orobanchaceae species.

Phylogenetic analyses of *atpH* genes from Orobanchaceae species including *A. indica* and other angiosperm species (Table S6) showed that *A. indica* is not closely related to Orobanchaceae species (Figure 3C). Park et al. [42] reported three chloroplast genes (*rps2, trnL-F,* and *rbcL*) in the genus *Phelipanche* (Orobanchaceae) were acquired from another Orobanchaceae species by HGT between chloroplast genomes. Our results suggest that the *atpH* gene in the *A. indica* chloroplast genome was acquired from another angiosperm chloroplast genome.

The *atpI* gene in *A. indica* mitogenome was also acquired from another angiosperm. This gene clustered closely with monocot species (Figure 3D), and monocots like *Miscanthus sinensis* are known *A. indica* hosts, which suggest that the *atpI* chloroplast gene was transferred from a host to *A. indica*. Most HGT events typically occur between mitochondrial or between chloroplast genomes of different species [8,42,43]. However, the *atpI* gene in the *A. indica* mitochondrial genome was acquired from the chloroplast genome of another species. Gandini and Sanchez-Puerta [9] suggested that native plastid sequences are initially transferred by IGT from plastids to mitochondria and then transferred to mitochondria of related species by HGT. We consider that the *atpI* of the *A. indica* mitogenome was introduced in the same manner.

### 2.4. Cytoplasmic Male Sterility (CMS) of Genes in the A. Indica Mitogenome

**Figure 4:**
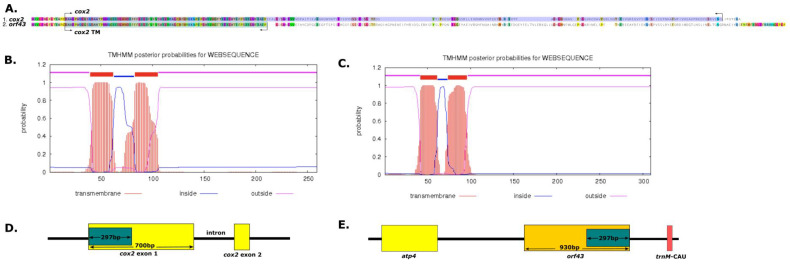
Comparison of *cox2* and *orf43* encoded proteins and prediction of transmembrane helices. (**A**) Comparison of the amino acids coded by *cox2* and *orf43*. (**B**) Prediction of transmembrane helices coded by *cox2*. (**C**) Prediction of transmembrane helices coded by *orf43*. (**D**) Location of the *cox2* gene in the *A. indica* mitogenome. (E) Location of the orf43 gene in the *A. indica* mitogenome. Small repeats that generate chimeric portions in the *cox2* xon1 and *orf 43* are shown in green (**D**,**E**). The arrow indicates each length.

Previous studies have shown that the production of functional pollen and structural variations in mt DNA are associated with CMS, which is caused by the expressions of chimeric open reading frames (ORFs) in the mitochondrial genome [12,15,44,45]. We identified 751 mitochondrial ORFs (≥150 bp in length) in *A. indica* and by BLAST searching *A. indica* mitochondrial genes. The *A. indica* mitogenome contained seven ORFs (≥30 bp in length), that is, *orf525, orf709 orf103, orf403, orf99, orf724,* and *orf 43* (Table S7). Of these, *orf43* contained fragments of *cox2* in the *A. indica* mitochondrial gene and was predicted to encode two transmembrane domains (Figure 4). Thus, *orf43* might be responsible for CMS. Previous studies have shown that the wild beet (*Beta vulgaris* ssp. *vulgaris*) [46], sunflower (*Helianthus annuus*) [16] and *Brassica* [47] mitogenomes contain two copies of *cox2* gene associated with CMS. Accordingly, our study provides clues regarding the evolution of CMS in Orobanchaceae.

## 3. Materials and Methods

### 3.1. Plant Samplingand DNA Sequencing

Orobanchaceae *Aeginatia indica* was collected from Jeju Island (Korea) and vouchers (YNUH-JAI001) were preserved in the herbarium of Yeungnam University. Genomic DNA was extracted from fresh leaf tissue using a DNeasy Plant Mini Kit (Qiagen, Hilden, Germany). Paired-end libraries with an average insert size of 550 bp using Illumina Hiseq 2500 (Illumina, San Diego, CA, USA). Approximately 20 Gb PE reads were generated.

### 3.2. Organellar Genome Assembly and Annotation

PE Illumina reads of genomic DNA were assembled de novo using Velvet v. 1.2.08 [48] and *k*-mers (69, 75, 81, 99, 105). Organellar contigs were identified in each assembly using blastn and known organellar gene sequences from Lamiales species. Organellar contgs from multiple assemblies were aligned manually in Geneious Prime v. 2021.0.3to construct the plastome and mitogenome of *A. indica*. To assess the depth of coverage, Illumina reads were mapped to the genome with Bowtie v 7.2.1 [49] in Geneious Prime. The *A. indica* plastome was annotated using DOGMA [50], Geseq [51], and Genious Prime, and the *A. indica* mitogenome was annotated using Mitofy [3], Geseq [51], and Genious Prime. All tRNA genes were predicted using tRNAscan-SE v.1.3.1 [52], and ORFs (>100 bp) were predicted and annotated using ORF-Finder in Geneious Prime. Plastome and mitochondrial genome maps were drawn using OGDRAW [53].

### 3.3. Plastome and Mitogenome Analyses

The complete plastome of *A. indica* (this study) was compared with that previously reported (MN529629) of *A. indica* [31] using the mVISTA program [54]. The *A. indica* plastome determined in the present study was used as a reference.

Tandem repeats in the mitochondrial genome were identified using Tandem Repeat Finder v4.04 [55]. Plastid-derived regions and plastid-like sequences transferred to mitochondrial genomes were estimated using cp genomes and BLASTN2.2.24+ using an e-value cutoff of 1 × 10^−6^ and at least 75% sequence identity.

### 3.4. Phylogenetic Analysis of Plastid and Mitochondrial Genes

Phylogenetic trees were constructed for: (1) 14 concatenated chloroplast genes of 34 species (Table S1) and (2) concatenated 19 mitochondrial genes of 20 species (Table S5). The 14 concatenated chloroplast genes of 34 species and the 19 concatenated mitochondrial genes of 20 species were aligned using MAFFT v7.222 [56]. Phylogenetic analyses were performed in RAxML using the GTRGAMMA model under rapid bootstrap values [57].

### 3.5. Analysis of Intracellular Gene Transfer (IGT), Horizontal Gene Transfer (HGT) and Cytoplasmic Male Sterility (CMS) Genes

IGT and HGT events in the *A. indica* mitogenome were identified using blastN and using an e-value cutoff of 1 × 10^−6^ searches for genes and ORFs of the mitochondrial genome against *Arabidopsis* plastid-encoded genes. For phylogenetic IGT and HGT analyses, sequenced mitochondrial and plastid genes across angiosperms were selected (Table S7). The data sets of individual mitochondrial and plastid genes were aligned using MAFFT [56] in Geneious Prime. Phylogenetic trees were constructed using RAxML and the GTRGAMMA model under rapid bootstrap values (1000 replicates) [57].

The ORFs of at least 150 bp were compared with identified *A. indica* mitochondrial genes using BlastN and an e-value cutoff of 1 × 10^−3^, a minimum length of 30 bp, and a sequence identity of at least 90%. Transmembrane domains in candidate ORFs were predicted using TMHMM v2.0 [58].

## Figures and Tables

**Table 1:** Gene contents of *A. indica* as determined in the previous study [MN52962] and this study. -; absent, o: present, (x2 ): numbers of duplication.

Category	Group of Gene	Genes	1 *	2 *	Category	Group of Gene	Genes	1 *	2 *
Photosynthethic	Phothsystem I	*psaA*	-	-	Self-replication	Large ribosomal subunit	*rpl2*	o(x2)	o
		*psaB*	-	-			*rpl14*	o(x2)	o
		*psac*	-	-			*rpl16*	o(x2)	o
		*psaI*	-	-			*rpl20*	o	o
		*psaJ*	-	-			*rpl22*	o(x2)	o
	Photosystem II	*psbA*	-	-			*rpl23*	o(x2)	-
		*psbB*	-	-			*rpl32*	-	-
		*psbC*	-	-			*rpl33*	o	o
		*psbD*	-	-			*rpl36*	o	o
		*psbE*	-	-		Small ribosomal subunit	*rps2*	o	o
		*psbF*	-	-			*rps3*	o(x2)	o
		*psbH*	-	-			*rps4*	o	o
		*psbI*	-	-			*rps7*	o(x2)	o
		*psbJ*	-	-			*rps8*	o(x2)	o
		*psbK*	-	-			*rps11*	o	o
		*psbL*	-	-			*rps12*	o(x2)	o
		*psbM*	-	-			*rps14*	o	o
		*psbN*	-	-			*rps15*	o	o
		*psbT*	-	-			*rps16*	o	o
		*psbZ*	-	-			*rps18*	o	o
	NADH dehydrogenase	*nadA*	-	-			*rps19*	o(x2)	o
		*ndhB*	ψ	ψ		RNA polymerase	*rpoA*	-	-
		*ndhC*	-	-			*rpoB*	-	-
		*ndhD*	-	-			*rpoC1*	-	-
		*ndhE*	-	-			*rpoC2*	-	-
		*ndhF*	-	-		ribosomal RNAs	*rrn23S*	o(x2)	o
		*ndhG*	-	-			*rrn16S*	o(x2)	o
		*ndhH*	-	-			*rrn5S*	o(x2)	o
		*ndhI*	-	-			*rrn4.5S*	o(x2)	o
		*ndhK*	-	-	Biosynthesis	Maturase	*matK*	o	o
	Cytochrome b/f complex	*petA*	-	-		Protease	*clpP*	o	o
		*petB*	-	-		Envelope membrane protein	*cemA*	-	-
		*petD*	-	-		Acetyl-CoA carboxylase	*accD*	o	o
		*petG*	-	-		cytochrome synthesis gene	*ccsA*	-	-
		*petL*	-	-		Translation initiation factor	*infA*	o	o
		*petN*	-	-	Unknown function	hypothetical chloroplast reading frame	*ycf1*	o	o
		*atpA*	-	-			*ycf2*	o	o
	ATP synthase Rubisco	*atpB*	-	-			*ycf3*	-	-
		*atpE*	-	-			*ycf4*	-	-
		*atpF*	-	-					
		*atpH*	-	o					
		*atpI*	-	-					
		*rbcL*	-	-					

1 *: Previous study [MN52962]. 2 *: This study.

## Data Availability

Not applicable.

## Supplementary Materials

The following are available online at https://www.mdpi.com/article/10.3390/ijms22116143/s1, Figure S1: Comparison of *Aeginetia indicla* plastid genome length and coverage between this study (A) and previous study (B). Figure S2: Comparison of the plastid genome sequences of this study and previous study generated using mVISTA program. Figure S3: Phylogenetic tree of 34 species (Table S1) taxa based on 14 chloroplast genes in the cp genome. Blue color indicates hemiparasite plants and red color indicates holoparsite plants in Orobanchacae. Figure S4: Coverage of *Aeginetia indica* mitogenome. Table S1: Source for plastid genomes included this study. Table S2: Blast result of plastid-derived DAN segments in mitochondrial genome of *Aeginetia indica* Gene contents of Angiosperm mitogenomes including *Aeginetia indica.* Table S3: *indica* Gene contents of Angiosperm mitogenomes including *Aeginetia indica.* Table S4: The tRNA gene contents of Angiosperm mitogenome including *Aeginetia indica.* Table S5: The intron contents of Angiosperm mitogenome including *Aeginetia indica.* Table S6: Soure for mitochondrial genoems included this study. Table S7: Taxon accession numbers for phylogenetic analysis of IGT and HGT. Table S8: Candidate CMS genes in *A. indica* mitochondrial genome.

## References

[1] Wicke S., Schneeweiss C.W., Depamphilis C.W., Mai F.M., Quandt D. (2011). The evolution of the plastid chromosome in land plants: Gene content, gene order, gene function. Plant Mol. Biol..

[2] Kubo T., Nishizawa S., Sugawara A., Otchoda N., Estiati A., Mikami T. (2000). The complete nucleotide sequence of the mitochondrial genome of sugar beet (*Beta vulagaris* L.) reveals a novel gene for tRNAcys (GCA). Nucleic Acids. Res..

[3] Alverson A.J., Wei X., Rice D.W., Stern D.B., Barry K., Palmer J.D. (2010). Insights into the evolution of mitochondrial genome size from complete sequences of *Citrullus lanatus* and *Cucurbita pepo* (Cucurbitaceae). Mol. Biol. Evol..

[4] Liao X., Zhao Y., Kong X., Khan A., Zhou B., Liu D., Kashif M.H., Chen P., Wang H., Zhou R. (2018). Complete sequence of kenaf (*Hibiscus cannabinus*) mitochondrial genome and comparative analysis with the mitochondrial genomes of other plants. Sci. Rep..

[5] Kan S.-L., Shen T.-T., Gong P., Ran J.-H., Wang X.-Q. (2020). The complete mitochondrial genome of *Taxus cuspidata* (Taxaceae): Eight protein coding genes have transferred to the nuclear genome. BMC Evol. Biol..

[6] Pinard D., Myburg A.A., Mizrachi E. (2019). The plastid and mitochondrial genomes of *Eucalyptus grandis*. BMC Genom..

[7] Raulet M.E., Garcia L.E., Gandini C.L., Sato H., Ponce G., Sanchez-Puerta M.V. (2020). Multichromosomal structure and foreign tracts in the *Ombrophytum subterraneum* (Balanophoraceae) mitochondrial genome. Plant Mol. Biol..

[8] Park S., Grewe F., Zhu A., Ruhlman T.A., Sabir J., Mower J.P., Jansen R.K. (2015). Dynamic evolution of Geranium mitochondrial genomes through multiple horizontal and intracellular gene transfers. New Phytol..

[9] Gandini C.L., Sanchez-Puerta M.V. (2017). Foreign plastid sequences in plant mitochondria are frequently acquired via mitochondrion-to-mitochondrion horizontal transfer. Sci. Rep..

[10] Zhao N., Wang Y., Hua J. (2018). The roles of mitochondrion in intragenomic gene transfer in plants: A source and a pool. Int. J. Mol. Sci..

[11] Dong S., Zhao C., Chen F., Liu Y., Zhang S., Wu H., Zhang L., Liu Y. (2018). The complete mitochondrial genome of the early flowering plant *Nymphaea colorata* is highly repetitive with low recombination. BMC Genom..

[12] Hanson M.R., Bentolila S. (2004). Interactions of mitochondrial and nuclear genes that affect male gametophyte development. Plant Cell..

[13] Liu H., Cui P., Zhan K., Lin Q., Zhuo G., Guo X., Ding F., Yang W., Liu D., Hu S. (2011). Comparative analysis of mitochondrial genomes between a wheat K-type cytoplasmic male sterility (CMS) line and its maintainer line. BMC Genom..

[14] Mower J.P., Case A.L., Floro E.R., Willis J.H. (2012). Evidence against equimolarity of large repeat arrangements and a predominant master circle structure of the mitochondrial genome from a monkeyflower (*Mimulus guttatus*) lineage with cryptic CMS. Genome Biol. Evol..

[15] Štorchová H., Stone J.D., Sloan D.B., Abeyawardana O.A.J., Müller K., Walterová J., Pažoutová M. (2018). Homologous recombination changes the context of Cytochrome b transcription in the mitochondrial genome of *Silene vulgaris* KRA. BMC Genom..

[16] Makarenko M.S., Usatov A.V., Tatarinova T.V., Azarin K.V., Logacheva M.D., Gavrilova V.A., Kornienko I.V., Horn R. (2019). Organization features of the mitochondrial genome of sunflower (*Helianthus annuus* L.) with ANN2-type male-sterile cytoplasm. Plants.

[17] Bennett J., Mathews S. (2006). Phylogeny of the parasitic plant family Orobanchaceae inferred from phytochrome A. Am. J. Bot..

[18] McNeal J.R., Bennett J.R., Wolfe A.D., Mathews S. (2013). Phylogeny and origins of holoparasitism in Orobanchaceae. Am. J. Bot..

[19] Shneeweiss G.M., Joel D.M., Gressel J., Musselman L.J. (2013). Phylogenetic relationships and evolution trends in Orobanchaceae. Parasitic Orobanchaceae: Parasitic Mechanisms and Control Strategies.

[20] Frailey D.C., Chaluvadi S.R., Vaughn J.N., Coatney C.G., Bennetzen J.L. (2018). Gene loss and genome rearrangement in the plastids of five hemiparasites in the family Orobanchaceae. BMC Plant Biol..

[21] Gruzdev E.V., Kadnikov V.V., Beletsky A.V., Mardanov A.V., Ravin N.V. (2019). Extensive plastome reduction and loss of photosynthesis genes in *Diphelypaea coccinea*, a holoparasitic plant of the family Orobanchaceae. Peer J..

[22] Wicke S., Müller J.F., de Pmphilis C.W., Quandt D., Wickett N.J., Zhang Y., Renner S.S., Schneeweiss G.M. (2013). Mechanisms of functional and physical genome reduction in photosynthetic and nonphotosynthetic parasitic plants of the broomrape family. Plant Cell..

[23] Fan W., Zhu A., Kozaczek M., Shah N., Pabón-Mora N., Gonzáles F., Mower J.P. (2016). Limited mitogenomic degradation in response to a parasitic lifestyle in Orobanchaceae. Sci. Rep..

[24] Zervas A., Petersen G., Severg O. (2019). Mitochondrial genome evolution in parasitic plants. BMC Evol. Biol..

[25] Mower J.P., Stefanović S., Young G.J., Palmer J.D. (2004). Gene transfer from parasitic to host plants. Nature.

[26] Li X., Zhang T.-C., Qiao Q., Ren Z., Zhao J., Yonezawa T., Hasegawa M., Crabbe M.J.C., Li J., Zhong Y. (2013). Complete chloroplast genome sequence of holoparasite *Cistanche deserticola* (Orobanchaceae) reveals gene loss and horizontal gene transfer from its host *Haloxylon ammodendron* (Chenopodiaceae). PLoS ONE.

[27] Kwolek D., Denysenko-Bennett M., Góralski G., Cygan M., Mizia P., Piwowarczyk R., Szklarczyk M., Joachimiak A.K. (2017). The first evidence of a host-to-parasite mitochondrial gene transfer in Orobanchaceae. Acta Biol. Cracoviensia Bot..

[28] Gandini C.L., Garcia L.E., Abbona C.C., Sanchez-Puerta M.V. (2019). The complete organelle genomes of *Physochlaina orientalis*: Insights into short sequence repeats across seed plant mitochondrial genomes. Mol. Phylognet. Evol..

[29] Kato Y., Inoue T., Onishi Y. (1984). In vitro culture of a root parasite, *Aeginetia indica* L. II. The plane of cell division in the tendril. Plant Cell Physiol..

[30] Li X., Feng T., Randle C., Schneeweiss G.M. (2019). Phylogenetic relationships in Orobanchaceae inferred from low-copy nuclear genes: Consolidation of major clades and identification of a novel position of the non-photosynthetic *Orobanche* clade sister to all other parasitic Orobanchaceae. Front. Plant Sci..

[31] Chen J., Yu R., Dai J., Liu Y., Zhou R. (2020). The loss of photosynthesis pathway and genomic locations of the lost plastid genes in a holoparasitic plant *Aeginetia indica*. BMC Plant Biol..

[32] Sloan D.B. (2013). One ring to rule them all? Genome sequencing provides new insights into the master cirlce model of plant mitochondrial DNA structure. New Phytol..

[33] Unseld M., Marienfeld J.R., Brandt P., Brennicke A. (1997). The mitochondrial genome of Arabidopsis thaliana contains 57 genes in 366,924 nucleotides. Nat. Genet..

[34] Asaf S., Khan A.L., Khan A.R., Waqas M., Kang S.-M., Khan M.A., Shahzad R., Seo C.-W., Shin J.-H., Lee I.-J. (2016). Mitochondrial genome analysis of wild rice (*Oryza minuta*) and its comparison with other related species. PLoS ONE.

[35] Adams K.L., Qiu Y.-L., Stoutemyer M., Palmer J.D. (2002). Punctuated evolution of mitochondrial gene content: High and variable rates of mitochondrial gene loss and transfer to the nucleus during angiosperm evolution. Proc. Natl. Acad. Sci. USA.

[36] Clifton S.W., Minx P., Fauron C.M., Gibson M., Allen J.O., Sun H., Tompson M., Barbazuk W.B., Kanuganti S., Tayloe C. (2004). Sequence and comparative analysis of the maize NB mitochondrial genome. Plant Physiol..

[37] Kubo T., Mikamu T. (2007). Organization and variation of angiosperm mitochondrial genome. Physiol. Plant..

[38] Grewe F., Zhu A., Mower J.P. (2016). Loss of a trans-splicing and1 intron from Geraniaceae and transfer of the maturase gene *matR* to the nucleus in *Pelargonium*. Genome Biol. Evol..

[39] Raman G., Park S., Lee E.M., Park S. (2019). Evidence of mitochondrial DNA in the chloroplast genome of *Convalaria keiskei* and its subsequent evolution in the Asparagales. Sci. Rep..

[40] Chen T.-C., Su Y.-Y., Wu C.-H., Liu Y.-C., Huang C.-H., Chang C.-C. (2020). Analysis of mitochondrial genomics and transcriptomics reveal abundant RNA edits and differential editing status in moth orchid, *Phalaenopsis aphrodite* subsp. *formosana*. Sci. Hortic..

[41] Cusimano N., Wicke S. (2016). Massive intracellular gene transfer during plastid genome reduction in nongreen Orobanchaceae. New Phytol..

[42] Park J.-M., Manen J.-F., Schneeweissm G.M. (2007). Horizontal gene transfer of a plastid gene in the non-photosynthetic flowering plants *Orobanche* and *Phelipanche* (Orobancheae). Mol. Phylogenet. Evol..

[43] Garcia L.E., Edera A.A., Palmer J.D., Sato H., Sanchez-Puerta M.V. (2021). Horizontal gene transfers dominate the functional mitochondrial gene space of a holoparastic plant. New Phytol..

[44] Abdelnoor R.V., Yule R., Elo A., Christensen A.C., Meyer-Gauen G., Mackenzie S.A. (2003). Substoichiometric shifting in the plant mitochondrial genome is influenced by a gene homologous to Muts. Proc. Natl. Acad. Sci. USA.

[45] Jo Y.D., Choi Y., Kim D.-H., Kum B.-D., Kang B.-C. (2014). Extensive structural variations between mitochondrial genomes of CMS and normal peppers (*Capsicum annuum* L.) revealed by complete nucleotide sequencing. BMC Genom..

[46] Yamamoto M.P., Shinada H., Onodera Y., Komaki C., Mikami T., Kubo T. (2008). A male sterility-associated mitochondrial protein in wild beets causes pollen disruption in transgenic plants. Plant J..

[47] Kang L., Li P., Wnag A., Ge X., Li Z. (2017). A novel cytoplasmic male sterility in Brassica napus (inap CMS) with carpelloid staens via protoplast fusion with Chinese woad. Font. Plant Sci..

[48] Zerbino D.R., Birney E. (2008). Velvet: Algorithms for de novo short read assembly using de Bruijn graphs. Genome Res..

[49] Langmead B., Salzberg S.L. (2012). Fast gapped-read alignment with Bowtie2. Nat. Methods.

[50] Wyman S.K., Jansen R.K., Boore J.L. (2004). Automatic annotation of organellar genomes with DOGMA. Bioinformatics.

[51] Tillich M., Lehwark P., Pellizzer T., Ulbricht-Jones E.S., Fischer A., Bock R., Greiner S. (2017). GeSeq-versatile and accurate annotation of organelle genomes. Nucleic Acids Res..

[52] Lowe T.M., Eddy S.R. (1997). tRNAscan-Se: A program for improved detection of transfer RNA genes in genomic sequence. Nucleic Acids Res..

[53] Lohse M., Drechsel O., Kahlau S., Bock R. (2013). OrganellarGenomeDRAW-a suite of tools for generating physical maps of plastid and mitochondrial genomes and visualizing expression data sets. Nucleic Acids Res..

[54] Frazer K.A., Pachter L., Poliakov A., Rubin E.M., Dubchak I. (2004). VISTA: Computational tools for comparative genomics. Nucleic Acids Res..

[55] Benson G. (1999). Tandem repeats finder: A program to analyze DNA sequences. Nucleic Acids Res..

[56] Katoh K., Misawa K., Kuma K., Miyata T. (2002). MAFFT: A novel method for rapid multiple sequence alignment based on fast Fourier transform. Nucleic Acids Res..

[57] Stamatakis A. (2006). RAxML-VI-HPC: Maximum likelihood-based phylogenetic analyses with thousands of taxa and mixed models. Bioinformatics.

[58] Krogh A., Larsson B., von Heijne G., Sonnhammer E.L. (2001). Predicting transmembrane protein topology with a hidden Markov model: Application to complete genomes. J. Mol. Biol..

