# Supplementary material for: Complete Plastid and Mitochondrial Genomes of Aeginetia indica Reveal Intracellular Gene Transfer (IGT), Horizontal Gene Transfer (HGT), and Cytoplasmic Male Sterility (CMS)
Source: Int J Mol Sci. 2021 Jun 7;22(11):6143. doi: 10.3390/ijms22116143 (PMC8201098; doi:10.3390/ijms22116143)
Supplement: Supplementary file 1 [file ijms-22-06143-s001.zip › ijms-1227376-supplementary.pdf]

Table S1. Source for plastid genomes included this study

Class	Order	Family	Species	Accession number
Asterids	Solanales	Solanaceae	*Solanum lycopersicum*	AC_000188
	Lamiales	Scrophulariceae	*Scrophularia takesimensis*	KM590983
		Lamiaceae	*Salvia miltorhhiza*	NC_023209
		Palowniaceae	*Paulownia coreana*	NC_031435
		Orobanchaceae	*Triaenophora shennongjiaensis*	NC_039781
			Rehmannia glutinosa	NC_034308
			Rehmannia elata	NC_034312
			Rehmannia chingii	NC_033534
			Lindenbergia phillippensis	NC_022859
			Aeginetia indica	MN529629
			***Aeginetia indica* (This study)**	**MW851293**
			Lathraea squamaria	NC_027838
			Brandisia swinglei	NC_042954
			Castilleja paramensis	NC_031806
			Pedicularis cheilanthifolia	NC_036010
			Pedicularis hallaisanensis	NC_037433
			Pedicularis ishidoyana	NC_029700
			Schwalbea americana	NC_023115
			Cistanche phelypaea	NC_025642
			Cistanche deserticola	NC_021111
			Epifagus virginiana	NC_001568
			Conopholis americana	NC_023131
			Phelipanche purpurea	NC_023132
			Orobanche californica	NC_025651
			Aphyllon epigalium subsp *epigalium*	MH050785
			Aphyllon fasciculatum	NC_039679
			Boulardia latisquama	NC_025641
			Orobanche rapum-genistae	NC_031444
			Orobanche pancicii	NC_031443
			Orobanche crenata	NC_024845
			Orobanche cernua var. *cumana*	KT387722
			Orobanche densiflora	NC_031442
			Orobanche austrohispanica	NC_031441
			Orobanche gracillis	NC_023464

Table S2. Blast result of plastid-derived DNA segments in mitochondrial genome of *Aeginetia indica*.

	Query start	Query end	Identity (%)	Alignment length	Subject start	Subject end	E value	Annotation
1	39,541	39,485	94.737	57	30,441	30,497	2.32E-18	*trnI-CAU*
2	39,609	39,538	98.611	72	30,369	30,440	1.63E-30	*rpl2/trnI-CAU IGS*
3	164,379	164,460	95.122	82	38,485	38,566	1.13E-31	*ndhB**
4	164,458	164,611	96.104	154	38,567	38,720	1.88E-70	*ndhB**
5	164,751	164,902	92.763	152	38,821	38,972	1.87E-59	*ndhB**
6	179,780	179,677	82.692	104	13,274	13,377	1.62E-19	*rps4***
7	179,993	179,922	91.667	72	12,422	12,493	7.82E-22	*trnS-GGA*
8	207,951	207,868	96.429	84	50,391	50,474	5.48E-34	*trnN-GUU*
9	247,816	247,739	91.026	78	15,230	15,307	1.44E-23	*trnM-CAU*
10	265,842	265,764	92.405	79	19,037	19,115	6.95E-26	*trnW-CCA*
11	273,732	273,793	96.774	62	43,250	43,311	5.44E-23	*rps7/rrn16 IGS*
12	393,071	392,994	92.308	78	9,450	9,527	2.63E-25	*trnD-GCU*

* indicate pseudogene, ** indicate fragments.

Table S3. The gene contents of Angiosperm mitogenome including *Aeginetia indica.* ●:present ○: absent

	Aeg	Cas	Mim	Sal	Rot	Aju	Hes	Nic	Sol	Rha	Ara	Sil	Gly	Med	Sen	Lac	Vit	Ory	Tri	Lir
atp1	●	●	●	●	●	●	●	●	●	●	●	●	●	●	●	●	●	●	●	●
atp4	●	●	●	●	●	●	●	●	●	●	●	●	●	●	●	●	●	○	●	●
atp6	●	●	●	●	●	●	●	●	●	●	●	●	●	●	●	●	●	●	●	●
atp8	●	●	●	●	●	●	●	●	●	●	●	●	●	●	●	●	●	○	●	●
atp9	●	●	●	●	●	●	●	●	●	●	●	●	●	●	●	●	●	●	●	●
atpI-cp	●	○	○	○	○	○	○	○	○	○	○	○	○	○	○	○	○	○	○	○
ccmB	●	●	●	●	●	●	●	●	●	●	●	●	●	●	●	●	●	●	●	●
ccmC	●	●	●	●	●	●	●	●	●	●	●	●	●	●	●	●	●	●	●	●
ccmFc	●	●	●	●	●	●	●	●	●	●	●	●	●	●	●	●	●	●	●	●
ccmFn	●	●	●	●	●	●	●	●	●	●	●	●	●	●	●	●	●	●	●	●
cob	●	●	●	●	●	●	●	●	●	●	●	●	●	●	●	●	●	●	●	●
cox1	●	●	●	●	●	●	●	●	●	●	●	●	●	●	●	●	●	●	●	●
cox2	●	●	●	●	●	●	●	●	●	●	●	●	●	○	●	●	●	●	●	●
cox3	●	●	●	●	●	●	●	●	●	●	●	●	●	●	●	●	●	●	●	●
matR	●	●	●	●	●	●	●	●	●	●	●	●	●	●	●	●	●	●	●	●
mttB	●	●	●	●	●	●	●	●	●	●	●	●	●	●	●	●	●	○	●	●
nad1	●	●	●	●	●	●	●	●	●	●	●	●	●	●	●	●	●	●	●	●
nad2	●	●	●	●	●	●	●	●	●	●	●	●	●	●	●	●	●	●	●	●
nad3	●	●	●	●	●	●	●	●	●	●	●	●	●	●	●	●	●	●	●	●
nad4	●	●	●	●	●	●	●	●	●	●	●	●	●	●	●	●	●	●	●	●
nad4L	●	●	●	●	●	●	●	●	●	●	●	●	●	●	●	●	●	●	●	●
nad5	●	●	●	●	●	●	●	●	●	●	●	●	●	●	●	●	●	●	●	●
nad6	●	●	●	●	●	●	●	●	●	●	●	●	●	●	●	●	●	●	●	●
nad7	●	●	●	●	●	●	●	●	●	●	●	●	●	●	●	●	●	●	●	●
nad9	●	●	●	●	●	●	●	●	●	●	●	●	●	●	●	●	●	●	●	●
rpl2	●	○	○	●	●	●	●	●	●	●	●		○	○	○	○	●	●	○	●
rpl5	●	●	●	●	●	○	●	○	●	●	●	●	●	●	●	●	●	●	●	●
rpl10	●	●	●	●	●	○	●	●	●	●	○	○	○	○	○	●	○	○	○	●
rpl16	●	●	●	●	●	○	●	●	●	●	●	○	●	●	●	●	●	○	●	●
rps1	○	○	○	○	○	○	○	●	●	●	○	○	●	●	●	○	●	●	●	●
rps2	○	○	○	○	○	○	○	○	○	○	○	○	○	○	○	○	○	●	●	●
rps3	●	●	●	●	●	●	●	○	●	●	●	●	●	●	●	●	●	●	●	●
rps4	●	●	●	●	●	●	●	○	●	●	●	●	●	●	●	●	●	●	●	●
rps7	○	○	○	●	○	○	●	○	○	●	●	○	○	○	○	○	●	●	●	●
rps10	●	●	●	●	●	○	●	●	●	●	○	○	●	●	●	○	●	○	○	●
rps11	○	○	○	○	○	○	○	○	○	○	○	○	○	○	○	○	●	○	○	●
rps12	●	●	●	●	●	●	●	○	●	●	●	○	●	●	●	●	●	●	●	●
rps13	●	●	●	●	●	●	●	●	●	●	○	○	○	○	○	●	●	●	○	●
rps14	●	●	●	●	●	○	●	●	○	●	○	●	●	●	●	○	●	○	○	●

rps19	○	○	○	○	○	○	○	●	●	●	○	○	○	○	○	○	●	●	●	●
sdh3	○	●	●	○	○	○	●	●	●	○	○	○	○	○	○	○	●	○	○	●
sdh4	ψ	○	●	○	○	○	●	●	●	●	○	○	○	○	○	○	●	○	○	●

Aeg=*Aeginetia indica*; Cas=*Castilleja paramensis*; Mim=*Mimulus guttatus*; Sal=*Salvia miltiorrhiza*; Rot=*Rotheca serrata*; Aju= *Ajuga reptans*; Hes=*Hesperelaea palmeri*; Nic=*Nicotiana tabacum*; Sol=*Solanum pennellii*; Rha=*Rhazya stricta*; Ara= *Arabidopsis thaliana*; Sil=*Silene latifolia*; Gly=*Glycine max*; Med=*Medicago truncatula*; Sen=*Senna tora*; Lac=*Lactuca sativa*; Vit= *Vitis vinifera*; Ory=*Oryza sativa*; Tri=*Triticum aestivum*; Lir=*Liriodendron tulipfera*

Table S4. The tRNA gene contents of Angiosperm mitogenome including *Aeginetia indica*. ●;present ○; absent ( ); numbers of duplication

	Aeg	Cas	Mim	Sal	Rot	Aju	Hes	Nic	Sol	Rha	Ara	Sil	Gly	Med	Sen	Lac	Vit	Ory	Tri	Lir
rrn5	●	●	●	●	●	●	●	●	●	●	●	●	●	●	●	●	●	●	●	●
rrn18	●	●	●	●	●	●	●	●	●	●	●	●	●	●	●	●	●	●	●	●
rrn26	●	●	●	●	●	●	●	●	●	●	●	●	●	●	●	●	●	●	●	●
trnA-UCU-CP	○	○	○	○	○	○	○	○	○	○	○	○	○	○	○	○	●	○	○	○
trnA-GUU-CP	○	○	○	○	○	○	○	○	○	○	○	○	○	○	○	○	●(3)	○	○	○
trnA-GUC-CP	○	○	○	○	○	○	○	○	○	○	○	○	○	○	○	○	●(2)	○	○	○
trnC-GCA	●	●(2)	●	●	●	●(2)	●	●	●	●	●	●	●	●	●	●	●	●	●	●
trnD-GCU-CP	●	●	●	●	●	●	●	●	○	●	○	○	●	●	●	○	○	●	●	○
trnD-GUC	○	○	○	○	○	○	○	○	●	○	●	○	○	○	○	●(2)	○		●	●
trnE-UUC	●	●	●	●	●	●	●	●(2)	●(2)	●	●	●	●	●	●	●	○	●	●	●
trnF-GAA	●	●(2)	●	●	●	●	●	●	●	●	○	○	●	●	●(2)	●	○	●	●	●
trnfM-CAU-CP	○	○	●	●	●	●	○	●(2)	○	●	●(2)	●	●(4)	●	●	○	●	●	○	●
trnG-GCC	○	○	●	●	○	○	●	●	○	●	●	○	●	●	●	●	●	○	○	●
trnG-UUC	○	○	○	○	○	○	○	○	○	○	○	○	○	○	○	○	●(2)	○	○	○
trnG-UUG	○	○	○	○	○	○	○	○	○	○	○	○	○	○	○	○	●	○	○	○
trnH-GUG	●	○	●	●	○	○	●	●	●	○	○	○	●	○	○	○	○	○	●	○
trnH-GUG-CP	○	●	○	○	○	○	○	○	○	●	●	●	○	●	●(2)	○	●	●	○	●
trnI-CAU	○	○	○	○	○	○	○	○	○	●	○	●	●	●	●	○	○	●	○	●(2)
trnI-CAU-CP	●	○	○	●(2)	●(2)	●(2)	●	●(2)	●(2)	●(2)	○	○	○	○	○	○	○	○	○	○
trnI-UAU-CP	○	○	○	○	○	○	○	○	○	○	○	○	○	○	○	○	●	○	○	○
trnK-UUU	●	●	●	●	○	○	●	●	●	●	●(2)	○	●	●	●(2)	●(2)	○	●	●(2)	●
trnL-CAA	○	●(3)	●	●	○	○	○	○	●	○	○	○	○	○	○	○	○	○	○	○
trnL-UAA	○	○	●	○	○	○	○	○	○	○	○	○	○	○	○	○	○	○	○	○
trnL-UAG-CP	○	○	○	○	○	○	○	○	○	○	○	○	○	○	○	○	●	○	○	○
trnL-UUU-CP	○	○	○	○	○	○	○	○	○	○	○	○	○	○	○	○	●	○	○	○
trnL-UUU	○	○	○	○	○	○	○	○	○	○	○	○	○	○	○	○	●	○	○	○
trnM-CAU	○	●(6)	●	●(2)	●(2)	●(2)	●	●	●(2)	○	●	○	●	●	●	●(5)	●	○	●(4)	●
trnM-CAU-CP	●	○	○	○	○	○	○	○	○	○	○	○	○	●	○	○	●	●	○	○
trnN-GUU	○	●	●	●	●	●	●	●	●(2)	●	●	●	●	○	●	○	○	○	○	○
trnN-GUU-CP	●	○	○	○	○	○	○	○	○	○	○	○	○	●	○	●	○	○	●	●
trnP-AGG-CP	○	○	○	○	○	○	○	○	○	○	○	●	○	○	○	○	○	○	○	○
trnP-GAA	○	○	○	○	○	○	○	○	○	○	○	○	○	○	○	○	●	○	○	○
trnP-UGG	●	●(2)	●	●	●	●	●	●	●(2)	●	●	●	●	●	○	●(2)	●	●	●(2)	●(3)
trnP-UGG-CP	○	○	○	○	○	○	○	○	○	○	○	○	○	○	○	○	●(2)	○	○	○
trnQ-UUG	○	●(2)	●	●	●	●	●	●	●	●	●	○	●	●	●	●	○	●	●(3)	●
trnR-ACG	○	○	○	○	○	○	●	○	○	○	○	○	○	○	○	○	○	○	○	○
trnR-UCU-CP	○	●	○	○	○	○	●	○	○	○	○	○	○	○	○	○	○	●	○	○

trnS-GCU	●	●-	●	●	●	●	●	●	●	●	●	○	●	○	●(2)	●(3)	●	○	○	●
trnS-GGA	●	●	●	●	○	○	●	●	●	●	●	○	○	○	○	○	●	●	●	○
trnS-UGA	○	○	○	●	○	●	●	●	●	●	●	○	○	○	○	●	●	○	○	●
trnT-CCA-CP	○	○	○	○	○	○	○	○	○	○	○	○	○	○	○	○	●(2)	○	○	○
trnT-GGU	○	●	●	○	○	○	○	○	○	○	○	○	○	○	○	●	●	○	○	○
trnT-GUA-CP	○	○	○	○	○	○	○	○	○	○	○	○	○	○	○	○	●	○	○	○
trnT-GUA	○	○	○	○	○	○	○	○	○	○	○	○	○	○	○	○	●	○	○	○
trnT-TGT	○	○	○	○	○	○	○	○	○	○	○	○	○	○	○	○	○	○	○	○
trnV-GAC-CP	○	○	○	●	○	○	○	○	○	●	○	○	○	○	●	○	○	○	○	○
trnV-TAC	○	○	○	○	○	○	○	○	○	○	○	○	○	○	○	○	○	○	○	●
trnW-CCA-CP	●	●	●	●	●	●	●	●	●	●	●	●	●	●	●	●	○	●	●	●
trnY-GUA	●	●	●	●	●	●	●	●	●	●	●(2)	●	●	●	●	●	○	●	●	●

Aeg=*Aeginetia indica*; Cas=*Castilleja paramensis*; Mim=*Mimulus guttatus*; Sal=*Salvia miltiorrhiza*; Rot=*Rotheca serrata*; Aju= *Ajuga reptans*; Hes=*Hesperelaea palmeri*; Nic=*Nicotiana tabacum*; Sol=*Solanum pennellii*; Rha=*Rhazya stricta*; Ara= *Arabidopsis thaliana*; Sil=*Silene latifolia*; Gly=*Glycine max*; Med=*Medicago truncatula*; Sen=*Senna tora*; Lac=*Lactuca sativa*; Vit= *Vitis vinifera*; Ory=*Oryza sativa*; Tri=*Triticum aestivum*; Lir=*Liriodendron tulipfera*

Table S5. The intron contents of Angiosperm mitogenome including *Aeginetia indica.* ●:present ○: absent, x: gene loss

	Aeg	Cas	Mim	Sal	Rot	Aju	Hes	Nic	Sol	Rha	Ara	Sil	Gly	Med	Sen	Lac	Vit	Ory	Tri	Lir
nad1-i394	●	●	●	●	●	●	●	●	●	●	●	●	●	●	●	●	●	●	●	●
nad1-i477	●	●	●	●	●	●	●	●	●	●	●	●	●	●	●	●	●	●	●	●
nad1-i669	●	●	●	○	●	●	●	●	●	●	●	●	●	●	●	●	●	●	●	●
nad1-i728	●	●	●	●	●	●	●	●	●	●	●	●	●	●	●	●	●	●	●	●
nad2-i156	●	●	●	●	●	●	●	●	●	●	●	●	●	●	●	●	●	●	●	●
nad2-i542	●	●	●	●	●	●	●	●	●	●	●	●	●	●	●	●	●	●	●	●
nad2-i709	●	●	●	●	●	●	●	●	●	●	●	●	●	●	●	●	●	●	●	●
nad2-i1282	●	●	●	●	●	●	●	●	●	●	●	●	●	●	●	●	●	●	●	●
nad4-i461	●	●	●	●	●	●	●	●	●	●	●	●	●	●	●	●	●	●	●	●
nad4-i976	●	●	●	●	●	●	●	●	●	●	●	○	●	●	●	●	●	●	●	●
nad4-i1399	●	●	●	●	●	●	●	●	●	●	●	●	●	●	●	●	●	●	●	●
nad5-i230	●	●	●	●	●	●	●	●	●	●	●	●	●	●	●	●	●	●	●	●
nad5-i1455	●	●	●	●	●	●	●	●	●	●	●	●	●	●	●	●	●	●	●	●
nad5-i1477	●	●	●		●	●	●	●	●	●	●	●	●	●	●	●	●	●	●	●
nad5-i1872	●	●	●	●	●	●	●	●	●	●	●	●	●	●	●	●	●	●	●	●
nad7-i140	●	●	●	●	●	●	●	●	●	●	●	●	●	●	●	●	●	●	●	●
nad7-i209	●	●	●	●	●	●	●	●	●	●	●	●	●	●	●	●	●	●	●	●
nad7-i676	○	○	○	○	○	○	○	○	○	○	●	●	●	●	●	●	●	●	●	●
nad7-i917	●	●	●	●	●	●	●	●	●	●	●	●	●	●	●	●	●	●	●	●
ccmFc-i829	●	●	●	●	●	●	●	●	●	●	●	●	●	●	●	●	●	●	●	●
cox1-i729	●	●	○	●	●	●	○	○	○	●	○	○	○	○	○	○	○	○	○	○
cox2-i373	○	○	○	○	○	○	○	○	●	○	○	○	○	○	○	○	●	●	●	●
cox2-i691	●	●	●	●	○	○	●	●	●	●	●	○	○	○	○	○	●	○	○	●
rpl2-i917	○	X	X	●	○	○	○	●	●	●	●	X	X	X	X	X	●	●	X	●
rps3-i74	●	●	●	●	●	○	●	●	X	●	●	○	●	●	●	●	●	●	●	●
rps10-i235	○	●	●	●	●	X	●	●	x	●	X	X	●	●	●	●	●	X	X	●

Aeg=*Aeginetia indica*; Cas=*Castilleja paramensis*; Mim=*Mimulus guttatus*; Sal=*Salvia miltiorrhiza*; Rot=*Rotheca serrata*; Aju= *Ajuga reptans*; Hes=*Hesperelaea palmeri*; Nic=*Nicotiana tabacum*; Sol=*Solanum pennellii*; Rha=*Rhazya stricta*; Ara= *Arabidopsis thaliana*; Sil=*Silene latifolia*; Gly=*Glycine max*; Med=*Medicago truncatula*; Sen=*Senna tora*; Lac=*Lactuca sativa*; Vit= *Vitis vinifera*; Ory=*Oryza sativa*; Tri=*Triticum aestivum*; Lir=*Liriodendron tulipfera*

Table S6. Source for mitochondrial genomes included this study.

Class	Order	Family	Species	Accession number
Magnoliopsida	Magnoliales	Magnoliaceae	*Liriodendron tulipfera*	NC_021152
Liliopsida	Poales	Poaceae	*Triticum aestivum*	NC_036024
			Oryza sativa	NC_011033
Rosids	Vitales	Vitaceae	*Vitis vinifera*	NC_012119
	Fabiales	Fabaceae	*Senna tora*	NC_038053
			Medicago truncatula	NC_029641
			Glycine max	NC_020455
Pentapetalae	Caryophyllales	Sileneae	*Silene latifolia*	NC_014487
Rosids	Brassicales	Brassicaceae	*Arabidopsis thaliana*	NC_037304
Asterids	Asterales	Asteraceae	*Lactuca sativa*	NC_042756
	Gentianales	Apocynaceae	*Rhazya stricta*	NC_024293
	Solanales	Solanaceae	*Nicotiana tabacum*	NC_006581
			Solanum pennellii	NC_-35964
	Lamiales	Oleaceae	*Hesperelaea paleri*	NC_031323
		Lamiaceae	*Salvia miltiorrhiza*	NC_023209
			Ajuga reptans	NC_023103
			Rotheca serrata	NC_049064
		Phrymaceae	*Mimulus guttatus*	NC_018041
		Orobanchaceae	*Castilleja paramensis*	NC_031806
			Aeginetia indica	**MW851294**

Table S7. Taxon accession numbers for phylogenetic analysis of IGT and HGT.

	Species	Accession number			
		cemA	*ndhB*	*atpH*	*atpI*
Amborellaceae	*Amborella trichopoda*	KF754803			
		o	o	o	o
Nymphaeaceae	*Nymphaea lotus*	NC_041238			
		o	o	o	o
Magnoliaceae	*Liriodendron chinense*	NC_030504			
		o	o	o	o
Araceae	*Spirodela polyrhiza*	NC_015891			
		o	o	o	o
Poaceae	*Elymandra subulata*	MH181214			
		o	o	o	o
Poaceae	*Miscanthus sinenis subsp. sinensis*	LN869228			
		o	o	o	o
Cephalotaceae	*Cephalotus follicularis*	NC_042597			
		o	x	o	o
Aquifoliaceae	*Ilex paraguariensis*	NC_031207			
		o	o	o	o
Aquifoliaceae	*Ilex latifolia*	NC_047291			
		o	o	o	o
Caprifoliaceae	*Heptacodium miconioides*	NC_042739			
		o	o	o	o
Caprifoliaceae	*Zabelia biflora*	NC_045063			
		o	o	o	o
Caprifoliaceae	*Zabelia triflora*	NC_045053			
		o	o	o	o
Oleaceae	*Fraxinus latifolia*	NC_042450			
		o	o	o	o
Plantaginaceae	*Triaenophora shennongjiaensis*	NC_039781			
		o	o	o	o
Orobanchaceae	*Rehmannia solanifolia*	NC_034310			
		o	o	o	o
Orobanchaceae	*Rehmannia glutinosa*	NC_034308			
		o	o	o	o
Orobanchaceae	*Rehmannia chingii*	NC_033534			
		o	o	o	o
Orobanchaceae	*Rehmannia henryi*	NC_034309			
		o	o	o	o
Orobanchaceae	*Rehmannia elata*	NC_034312			
		o	o	o	o
Orobanchaceae	*Rehmannia piasezkii*	NC_034311			
		o	o	o	o
Orobanchaceae	*Siphonostegia chinensis*	NC_046038			
		o	o	o	o
Orobanchaceae	*Schwalbea americana*	NC_023115			
		o	o	o	o
Orobanchaceae	*Brandisia swinglei*	NC_042954			
		o	o	o	o
Orobanchaceae	*Lathraea squamaria*	NC_027838			
		x	x	o	o
Orobanchaceae	*Euphrasia regelii*	NC_045041			
		x	x	o	o
Orobanchaceae	*Melampyrum roseum*	MN075942			
		o	o	o	o
Orobanchaceae	*Lindenbergia philippensis*	NC_022859			
		o	o	o	o

Orobanchaceae	*Orobanche californica*	NC_025651				
			x	x	x	o
Orobanchaceae	*Phelipanche purpurea*	NC_023132				
			x	x	o	o
Orobanchaceae	*Phelipanche aegyptiaca*	KU21370				
			x	x	o	o
Orobanchaceae	*Phelipanche ramose*	NC_023465				
			x	x	o	o

The present of the each gene in each plastid genome is indicated with an "o"
The absence of the each gene in each plastid genome is indicated with an "x"

Table S8. Candidate CMS genes in *A. indica* mitochondrial genome.

	ORF start	ORF end	ORF length	Identity	ORF hit start	ORF hit end	Chimera length	Gene hit start	Gene hit end	E-value	Gene	No of Transmembrane helices / probabilities
ORF525	358,448	358,266	183	96.774	151	181	31	1,554	1,584	1.08e-08	*rps3*	0/0.67375
ORF709	204,364	204,095	270	95.349	133	175	43	868	910	4.52e-14	*atp6*	2/0.41976
ORF103	289,222	289,401	180	95	126	87	40	538	577	3.81e-12	*ccmFn*	0/0.39590
ORF403	347,931	347,707	225	96.667	111	82	30	548	577	4.30e-08	*ccmFn*	0/0.34781
ORF99	282,157	282,414	258	92.308	48	10	39	588	626	7.22e-10	*cox1*	0/0.22222
ORF724	137,638	137,480	159	94.286	106	72	35	151	185	2.74e-09	*cox1*	0/0.47309
ORF43	338,288	339,217	930	100	1	298	298	1	298	2.71e-165	*cox2*	2/0.00547

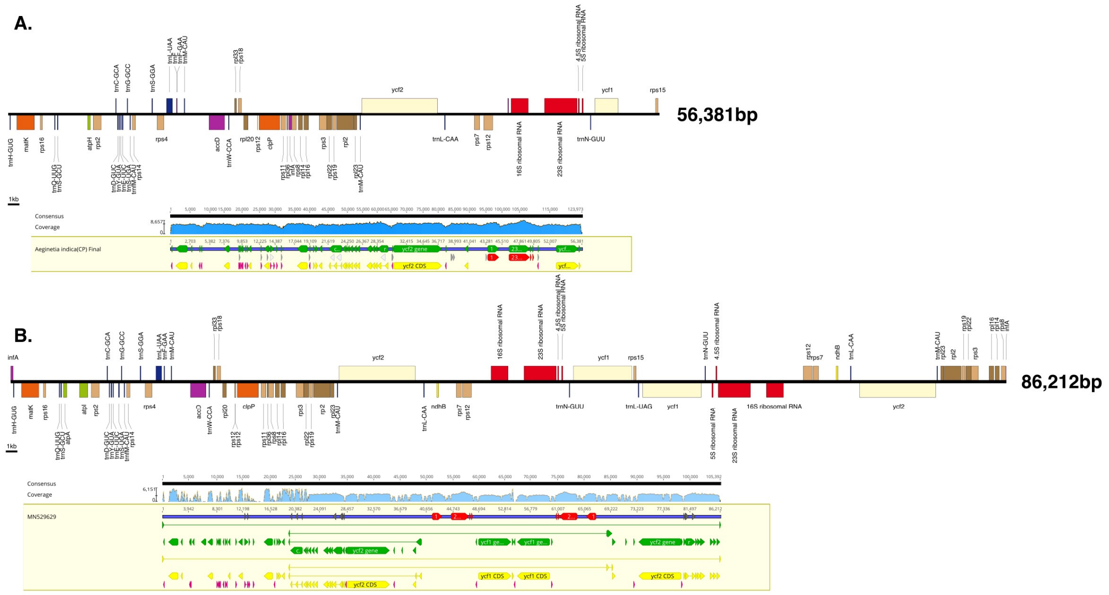

Figure S1

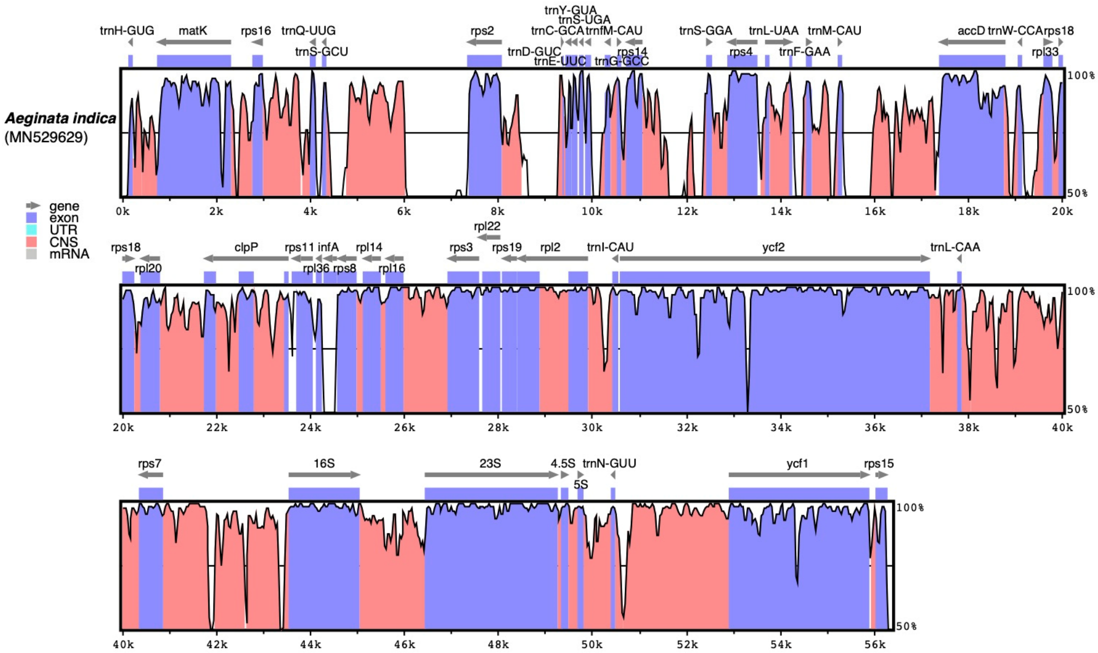

Figure S2

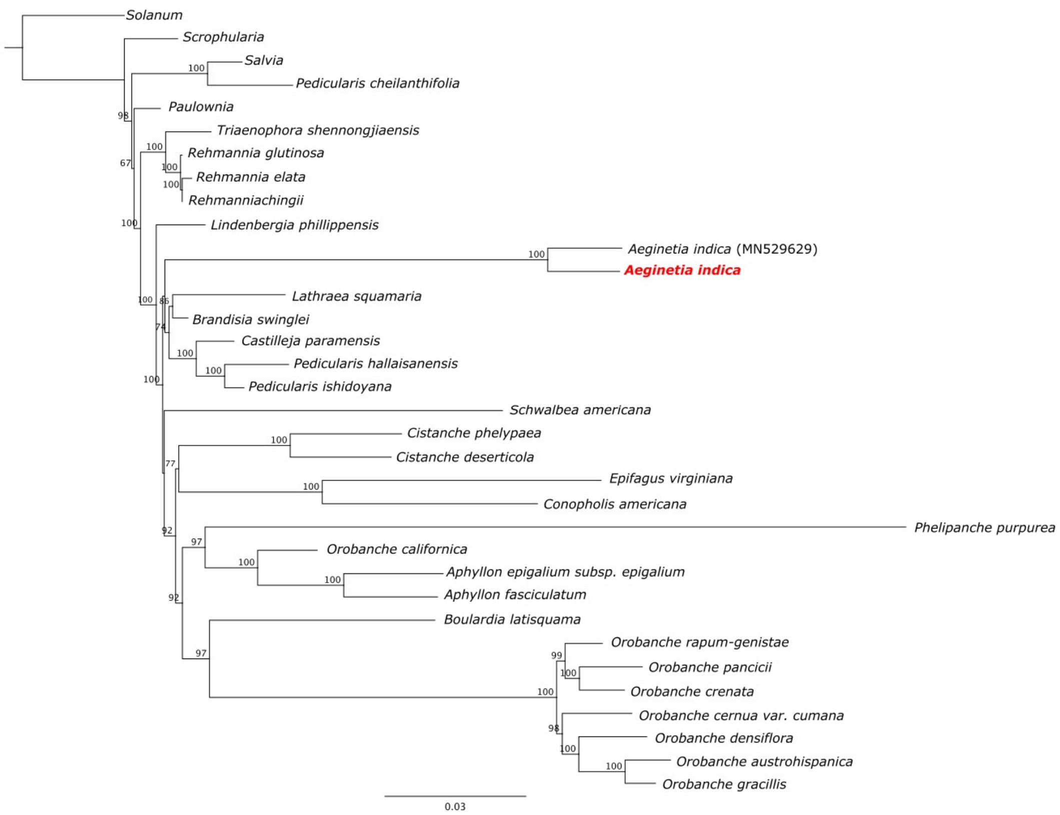

Figure S3

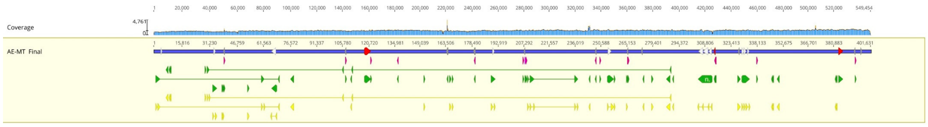

Figure S4
